# Impact of porin deletions on cefepime-taniborbactam activity against *Klebsiella pneumoniae*

**DOI:** 10.1128/aac.01672-24

**Published:** 2025-04-02

**Authors:** Tsuyoshi Uehara, Cassandra L. Chatwin, Brittany Miller, Mitchell Edwards, Annie Stevenson, Jenna Colombo, Kyoko Uehara, Denis M. Daigle

**Affiliations:** 1 Venatorx Pharmaceuticals Inc. 540451, Malvern, Pennsylvania, USA; University of Pittsburgh School of Medicine, Pittsburgh, Pennsylvania, USA

**Keywords:** cefepime-taniborbactam, NDM-1, *Klebsiella pneumoniae*, porins

## Abstract

We determined the frequency of resistance to cefepime-taniborbactam in NDM-1-producing *Klebsiella pneumoniae* at <10^−10^. Isolated mutants that were less susceptible to cefepime-taniborbactam had an increased copy number of the *bla*_NDM-1_ gene or disruption of major porins OmpK36 and OmpK35. Antibacterial susceptibility testing using *K. pneumoniae* isogenic strains indicated that while cefepime penetrates into the periplasm mainly through the major porins, taniborbactam does not have a strong dependence on OmpK35 or OmpK36 for periplasmic accumulation.

## INTRODUCTION

Cefepime-taniborbactam is a novel β-lactam-β-lactamase inhibitor (BL-BLI) combination to treat complicated urinary tract infections associated with multidrug-resistant pathogenic Gram-negative bacteria (1–3). In the CERTAIN-1 phase 3 clinical trial, cefepime-taniborbactam was statistically superior to meropenem for the primary composite endpoint at the test of cure visit (4, 5). Cefepime is a fourth-generation cephalosporin that inhibits the growth of susceptible bacteria by covalently binding to penicillin-binding proteins (PBPs) required for the synthesis of the bacterial cell wall (6). Taniborbactam is a broad-spectrum cyclic boronate BLI that inhibits all classes of β-lactamases, including extended-spectrum β-lactamases and carbapenemases, thereby restoring the activity of cefepime against multidrug-resistant Enterobacterales and *Pseudomonas aeruginosa* (1, 2). Although taniborbactam is a potential inhibitor of NDM-1 (2), recent reports describe two NDM variants, NDM-9 and NDM-30, that are not inhibited by taniborbactam (7–10). In addition, VIM-83, a VIM-1-like β-lactamase, is poorly inhibited by taniborbactam (11). Among 20,725 Enterobacterales and 7,919 *P*. *aeruginosa* clinical isolates tested in the GEARS global surveillance program, only one isolate carried NDM-9 (*K. pneumoniae* from the Philippines) while neither NDM-30 nor VIM-83 was identified (12). The potential for the emergence of NDM-9 is concerning as it is a cefepime-taniborbactam-resistant variant of NDM-1 mediated by a single-nucleotide mutation that codes for a Glu152 to Lys (codon changed from GAG to AAG) substitution. However, the frequency at which these escape variants resistant to taniborbactam would be developed from an NDM-1-producing strain has not been determined.

To determine the frequency of resistance (FoR) to cefepime-taniborbactam in *K. pneumoniae* producing NDM-1, we used the OMAN 8 and 19 strains (kindly provided by Dr. Patrice Nordmann, University of Fribourg) (13). The procedure of the FoR experiment is described in Supplemental Material and Table S1. The agar MICs of cefepime with 4 µg/mL taniborbactam against both isolates were 4 µg/mL (Table S1). Approximately 10^11^ cells were plated on agar containing 16 µg/mL cefepime and 4 µg/mL taniborbactam (4 × agar MIC for cefepime). After incubation for 48 hours at 37°C, five and three colonies arose for OMAN 8 and 19, respectively. Accordingly, the frequencies of colonies arising on agar containing 4 × MIC of cefepime-taniborbactam were low, at 5.0 × 10^−11^ for OMAN 8 and 3.3 × 10^−11^ for OMAN 19 (Table 1). The low FoR is consistent with previous observations against carbapenem-producing Enterobacterales (2). After isolated colonies from *K. pneumoniae* OMAN 8 and 19 from agar plates were grown under antibiotic selection, broth microdilution susceptibility assays confirmed that these colonies were less susceptible to cefepime-taniborbactam than the parent strains (Table 1). The cefepime-taniborbactam MIC was 16 µg/mL against a mutant derived from OMAN 8 and 64 µg/mL against a mutant derived from OMAN 19, while the cefepime-taniborbactam MIC was 2 and 1 µg/mL against the OMAN 8 and 19 parent strains, respectively. Susceptibility to cefepime alone was decreased fourfold for mutants compared to the parent strains and was consistent with the elevated MICs of meropenem. Taniborbactam fixed at 4 µg/mL decreased the meropenem MIC to 0.25 µg/mL in both parent strains, while in OMAN 8 and 19 mutants, the meropenem-taniborbactam MICs were 8 µg/mL and 128 µg/mL, respectively. The levels of potentiation of the cefepime and meropenem MICs by the addition of taniborbactam were similar in the OMAN 8 mutant compared to the parent, whereas it was reduced in the OMAN 19 mutant relative to the parent. This suggests that taniborbactam retains inhibitory activity against NDM-1 and other β-lactamases produced in the OMAN 8 mutant, while cefepime and meropenem are substantially inactivated even in the presence of taniborbactam in the OMAN 19 mutant.

**TABLE 1:** Genome sequence analysis of the parent and mutant strains^*a*^

Strain/ mutant	Frequency of resistance	Modal broth MIC (µg/mL)^*b*^				WGS analysis^*d*^						β-lactamase activity±SD^*g*^ (nmol/min/mg protein)	
		FEP	FEP+TAN^*c*^	MEM	MEM+TAN^*c*^	ST type	β-lactamase identified	*bla*_NDM-1_ fold coverage^*e*^	*bla*_OXA-1_ fold coverage^*d*^	OmpK35	OmpK36	Untreated	+ 4 µg/mL avibactam
OMAN 8	4.95 × 10^−11^	32	2	32	0.25	340	NDM-1 OXA-1 SHV-11	1.7	1.0	No lesion	No lesion	22.8 ± 3.4	10.5 ± 1.5
OMAN 8 mutant #1	NA	128	16	128	8	340	No change	**14.7** ^*h*^	0.9	No change	No change	68.9 ± 2.3	**52.2 ± 7.9**
OMAN 19	3.33 × 10^−11^	128	1	64	0.25	15	NDM-1 OXA-1 CTX-M-15 SHV-12 SHV-28	1.7	0.4	**Lesion** (**W230 stop**)	No lesion	369 ± 181	23.2 ± 9.7
OMAN 19 mutant #1	NA	512	64	512	128	15	No change	2.5	0.1	No change	**Lesion^*f*^**	396 ± 178	23.6 ± 7.9

^*a*^ ST, serotype; NA, not applicable.

^*b*^ MIC determination was performed according to CLSI recommendations (14). The modal MICs shown were determined from five independent experiments. The raw MIC data against QC strains were in the QC range (15).

^*c*^ Taniborbactam (TAN) was tested at a fixed concentration of 4 μg/mL in combination with cefepime (FEP) and meropenem (MEM).

^*d*^ In every strain, no lesion was found in the *ramR* gene and the *ftsI* gene encoding PBP3 was wild type.

^*e*^ Fold coverage is the fold change of the average coverage in the β-lactamase open reading frame to the average coverage of the genome.

^*f*^ Lesion was a frameshift mutation caused by a 2-base-pair deletion at codons 159–160.

^*g*^ β-lactamase activity in cell extracts was measured using nitrocefin in the absence or presence of 4 µg/mL avibactam in three independent experiments. Avibactam was used to inhibit serine β-lactamases in the cell extract to measure NDM-1 activity. SD, standard deviation. The methods are described in Supplemental Material.

^*h*^ Changes that would increase the MIC of cefepime-tanibobactam are shown in bold.

To identify the mechanism of resistance to cefepime-taniborbactam in the mutants isolated spontaneously by selection, whole genome sequencing analysis of the parent and mutant strains was performed as described in Supplemental Material. Using multilocus sequence typing, the sequence types of OMAN 8 and 19 for both parent and mutant strains were 340 and 15, respectively (Table 1). Using a computational tool to identify β-lactamases in assembled contigs, we found that OMAN 8 carried genes encoding NDM-1 as well as OXA-1 and SHV-11, while OMAN 19 possessed genes producing NDM-1, OXA-1, CTX-M-15, SHV-12, and SHV-28. Attempts to identify any unique mutation in the OMAN 8-mutant genome compared to the parent genome did not yield any convincing base-pair variation. No sequence changes were found in the genes encoding NDM-1, OXA-1, or SHV-11. However, the mutant genome showed a 14.7-fold higher coverage of the *bla*_NDM-1_ gene than the parent genome, suggesting multiplication of the NDM-1 gene that would lead to overexpression of NDM-1. This is supported by the observation that the cell extract prepared from the mutant cells had fivefold higher β-lactamase activity than that from the parent when serine β-lactamases were inhibited by avibactam (Table 1). Previously, the existence of a DNA unit containing *bla*_NDM-1_ in the form of tandem repeats has been reported in a clinical isolate of multidrug-resistant *K. pneumoniae* (16). This is a plausible explanation for these elevated MICs of cefepime, cefepime-taniborbactam, meropenem, and meropenem-taniborbactam.

By comparing the mapped reads of the OMAN 19 parent and mutant to the reference genome (see Supplementary Material), a frameshift mutation in the *ompK36* gene (caused by a 2-base pair deletion at codons 159–160) was identified only in the mutant (Table 1). OmpK36 and OmpK35 are major outer membrane porins, and loss of OmpK36 and OmpK35 reduces susceptibility to many β-lactams including cefepime and meropenem as well as other classes of antibiotics in *K. pneumoniae* by decreasing drug penetration through the outer membrane into the periplasm where the PBPs are located (17–19). Indeed, the *ompK35* gene had already been disrupted in the parent by a nonsense mutation at codon 230, and the nonsense mutation was maintained in the mutant. This resulted in the deletion of both major porins in the mutant, which likely decreases the cellular accumulation of cefepime, thus reducing susceptibility to cefepime-taniborbactam. This is consistent with the previous observation that the deletion of the major porins mediates the elevated MIC of cefepime-taniborbactam in *K. pneumoniae*-producing NDM or VIM metallo-β-lactamases (12, 20–22). We also examined the *ftsI* gene encoding PBP3, which is the major target of cefepime (6) as well as the *ramR* gene, as overexpression of the AcrAB-TolC efflux pump by *ramR* deletion is implicated in reducing the activity of β-lactams (23). No mutation was found in the genes encoding PBP3 and RamR. Importantly, no sequence changes were found in the genes encoding NDM-1, OXA-1, CTX-M-15, SHV-12, or SHV-28.

To further examine the impact of porin deletions on the *in vitro* activity of cefepime-taniborbactam in *K. pneumoniae*, we determined the MIC of antibiotics against isogenic strains lacking OmpK35 and/or OmpK36 and both deleted along with RamR (Kemyth Biotech, Taiwan) (18). The single deletion of either OmpK35 or OmpK36 had no effect on, or only slightly decreased, the antibacterial activities of β-lactams tested (cefepime, ceftazidime, meropenem, aztreonam, and cefoxitin), with ≤4-fold increase in MIC while having no effect on the chloramphenicol MIC (Table 2). The dual deletion of OmpK35 and OmpK36 (strain C13) increased the MIC by 4- to 32-fold for all β-lactams. The loss of *ramR* (strain C14), which induces overexpression of the AcrAB efflux pump (23, 24), reduced susceptibility to chloramphenicol, cephalosporins, and aztreonam but had no effect on susceptibility to meropenem. Cells lacking OmpK35, OmpK36, and RamR (strain C17) showed the highest MIC for every antibiotic tested, suggesting that porin deletion in concert with efflux overexpression decreases the cellular accumulation of these antibiotics.

**TABLE 2:** Impact of porin deletion and AcrAB-TolC efflux pump overexpression of the activity of antibiotics including cefepime-taniborbactam^*a*^^,^^*b*^

Strain	Strain description	Modal broth MIC (µg/mL)										
		Cefepime				Ceftazidime		Meropenem		ATM	FOX	CHL
			+TAN	+AVI	+VAB		+AVI		+VAB			
C01	NVT1001, parental strain	0.06	0.06	0.03	0.03	0.12	0.12	0.03	0.03	≤0.06	2	8
C11	Δ*ompK35*	0.06	0.06	0.06	0.06	0.25	0.25	0.03	0.03	0.12	8	8
C12	Δ*ompK36*	0.06	0.12	0.06	0.12	0.12	0.12	0.03	0.03	≤0.06	8	8
C13	Δ*ompK35* Δ*ompK36*	0.25	0.5	0.5	0.5	0.5	0.5	0.25	0.25	0.25	64	8
C14	Δ*ramR*	0.25	0.25	0.25	0.25	1	0.5	0.03	0.03	0.25	64	64
C17	Δ*ompK35* Δ*ompK36* Δ*ramR*	2	2	1	2	2	1	0.5	0.5	0.5	256	128
C01-P132	NVT1001/KPC-3	512	0.03	0.06	0.06	128	2	16	0.03	>128	16	4
C11-P132	Δ*ompK35*/KPC-3	512	0.06	0.12	0.12	512	4	128	0.03	>128	64	8
C12-P132	Δ*ompK36*/KPC-3	1024	0.12	0.25	0.25	256	2	32	0.06	>128	64	4
C13-P132	Δ*ompK35* Δ*ompK36*/KPC-3	>1024	1	2	2	512	8	512	32	>128	256	8
C14-P132	Δ*ramR* / KPC-3	1024	0.25	0.25	0.25	512	4	64	0.06	>128	64	64
C17-P132	Δ*ompK35* Δ*ompK36* Δ*ramR* / KPC-3	>1024	4	4	8	>1024	16	1024	32	>128	512	64

^*a*^ Taniborbactam (TAN) and avibactam (AVI) were tested at a fixed concentration of 4 μg/mL each in combination with cefepime and ceftazidime. Vaborbactam (VAB) was tested at a fixed concentration of 8 μg/mL in combination with cefepime and meropenem. The modal MICs shown were determined from five independent experiments.

^*b*^ TAN, taniborbactam; AVI, avibactam; VAB, vaborbactam; ATM, aztreonam; FOX, cefoxitin; CHL, chloramphenicol.

In the *K. pneumoniae* parental strain, the expression of KPC-3 β-lactamase raised the cefepime MIC from 0.06 to 512 µg/mL. With KPC-3 expressed, the cefepime MIC was further elevated twofold by the loss of OmpK36 or RamR and >2-fold by the loss of both porins (from 512 µg/mL to >1024 µg/mL), while it was not impacted by the loss of OmpK35. As fourfold variations in MIC (i.e., ±1 doubling dilutions) are within the acceptable inherent variability of the MIC assay (14), the effect of porin deletion on cefepime activity in KPC-3-producing strains is considered minimal. The meropenem MIC was elevated by the addition of carbapenemase KPC-3 to 16 µg/mL and further elevated 32-fold by the loss of both porins and 4-fold by the loss of RamR. The increase in the meropenem MIC by the porin deletion was greater in strains expressing KPC-3 relative to the parent β-lactamase-negative strain, suggesting a synergistic impact of porin deletion and β-lactamase on meropenem activity. This synergistic behavior was not detected for cefepime as the MICs were already high in the parent strain expressing KPC-3. The addition of taniborbactam, avibactam, or vaborbactam decreased the cefepime MIC substantially in all strains expressing KPC-3, consistent with their inhibitory activity of KPC-3. The result also shows that these three BLIs accumulate sufficiently to inhibit KPC-3 in the parent strain.

The cefepime MIC against the KPC-3-producing strain lacking both porins (strain C17-P132) and RamR was >1,024 µg/mL and was reduced to 4 µg/mL in the presence of taniborbactam, 4 µg/mL in the presence of avibactam, and 8 µg/mL in the presence of vaborbactam. Regardless of the loss of porins and/or RamR, the cefepime MICs in the presence of these BLIs were similar against strains producing KPC-3 as in the corresponding strains lacking the carbapenemase (≤4-fold MIC changes). These results indicate that while intracellular levels of cefepime decrease by loss of porins and RamR, these BLIs accumulate sufficiently to inhibit KPC-3 in these mutants. In contrast to cefepime-avibactam, the ceftazidime-avibactam MIC was elevated at least eightfold by the expression of KPC-3 regardless of mutations in the porin genes and/or *ramR*, indicating that the loss of porins and deletion of *ramR* reduce ceftazidime activity more than cefepime. Meropenem and meropenem-vaborbactam were most impacted by the deletion of the porins consistent with previous findings (25). The meropenem-vaborbactam MIC increased 1,024-fold by the porin deletion in strains expressing KPC-3 (0.03 to 32 µg/mL), while the corresponding elevation of meropenem MIC was 32-fold (16 to 512 µg/mL), suggesting that intracellular accumulation of vaborbactam is reduced by deletion of both porins. Notably, the Δ*ompK35* Δ*ompK36* Δ*ramR* mutant expressing KPC-3 was more susceptible to cefepime-taniborbactam (MIC, 4 µg/mL) than ceftazidime-avibactam or meropenem-vaborbactam (MIC, 16 and 32 µg/mL, respectively).

In summary, the FoRs to cefepime-taniborbactam in two *K. pneumoniae* strains producing NDM-1 were low (approximately 5 × 10^−11^). In isolated mutants that were less susceptible to cefepime-taniborbactam, taniborbactam remained able to reduce the cefepime MIC. Multiplication of the *bla*_NDM-1_ gene likely contributed to the elevated MIC of cefepime-taniborbactam in one mutant (OMAN 8). The loss of both major porins (OmpK36 and OmpK35) decreased susceptibility to cefepime-taniborbactam in the other mutant (OMAN 19), likely owing to reduced cellular accumulation of cefepime. MIC data in the isogenic *K. pneumoniae* strains suggest that the loss of both major porins reduces the cellular accumulation of cefepime, ceftazidime, meropenem, and vaborbactam, while it does not affect the accumulation of taniborbactam and avibactam. Overexpression of the AcrAB-TolC efflux pump by deletion of the *ramR* regulatory gene had little impact on the activities of the β-lactams and the BL-BLI combinations tested in this study. Notably, no nucleotide substitution in β-lactamase genes was found in the FoR study, especially those that would convert NDM-1 to NDM-9, suggesting a low probability that clinical use of cefepime-taniborbactam would accelerate the emergence of NDM-9.

## Data Availability

Raw sequence reads of the OMAN 8 and 19 parent and mutant strains were deposited in GenBank under BioProject accession number PRJNA1232128.

## SUPPLEMENTAL MATERIAL

Supplemental material aac.01672-24-s0001.pdf Supplemental methods; Tables S1 and S2.

ASM does not own the copyrights to Supplemental Material that may be linked to, or accessed through, an article. The authors have granted ASM a non-exclusive, world-wide license to publish the Supplemental Material files. Please contact the corresponding author directly for reuse.
