# Supplementary material for: Impact of porin deletions on cefepime-taniborbactam activity against Klebsiella pneumoniae
Source: Antimicrob Agents Chemother. 2025 Apr 2;69(5):e01672-24. doi: 10.1128/aac.01672-24 (PMC12073839; doi:10.1128/aac.01672-24)
Supplement: Supplemental material — Supplemental methods; Tables S1 and S2. [file aac.01672-24-s0001.pdf]

Supplemental Material

Impact of porin deletions on cefepime-taniborbactam activity against *Klebsiella pneumoniae*

Tsuyoshi Uehara*, Cassandra L. Chatwin, Brittany Miller, Mitchell Edwards, Annie Stevenson, Jenna Colombo, Kyoko Uehara, and Denis M. Daigle

Venatorx Pharmaceuticals, Inc. Malvern, Pennsylvania, United States

Short running title: Impact of porin deletion on cefepime-taniborbactam

***Correspondence to:** uehara@venatorx.com

Keywords: Cefepime-taniborbactam, NDM-1, *K. pneumoniae*, porins

Materials and Methods

Antibiotics

Cefepime (catalog #: 1097636), meropenem (catalog #: 1392454), and aztreonam (catalog #: 1046205) were purchased from USP (Rockville, MD). Ceftazidime (catalog #: A6987-1G), chloramphenicol (catalog #: C0857-25G) and kanamycin (catalog #: K1637-25G) were purchased from Sigma Aldrich (St. Lous, MO). Cefoxitin (catalog #: J66800) was purchased from Alfa Aesar (Tewksbury, MA). Taniborbactam (lot #: DMG00039.169.1) was synthesized by Venatorx. Avibactam (lot #: NJL-159-054-4) was prepared from Avycaz as described previously (1). Vaborbactam (lot #: RT00097-129) was prepared from a vial of Vabomere by Venatorx.

Bacterial strains and plasmids

The NDM-1-producing *Klebsiella pneumoniae* strains used in this study (strains OMAN 8 and OMAN 19) were obtained from Professor Patrice Nordmann at the University of Fribourg. The strain information is described previously (2). The *K. pneumoniae* NVT1001 strain (capsular serotype 1) and isogenic mutants lacking OmpK35, OmpK36 and/or RamR were obtained from Kemyth Biotech Company (www.kemyth.com, New Taipei City, Taiwan). Detailed information on the strains was described previously (3). The strains expressing KPC-3 carbapenemase from the kanamycin-resistant pACYC177 plasmid were used to assess activity of β-lactamase inhibitors.

In vitro Susceptibility Testing

The in vitro antibacterial activity of β-lactams alone or in combination with β-lactamase inhibitors was determined in cation-adjusted Mueller Hinton broth (CAMHB) microdilution assays according to CLSI recommendations (4). The potentiation of cefepime antibacterial activity by taniborbactam was assessed by fixing the BLI concentration at 4 μg/mL, unless otherwise stated (5). The inocula for the broth microdilution assays were prepared by the broth culture method (4). The β-lactams were 2-fold serially diluted in CAMHB with or without BLI. The minimum inhibitory concentration

(MIC) was defined as the lowest concentration of a β-lactam that inhibited the visible growth of parent and mutant strains after overnight incubation. Similarly to the broth dilution MIC method described above, agar MIC was performed on Mueller Hinton broth containing 1.5% agar (MHA) and antibiotics tested with an inoculum of 10^4 CFU per spot. Agar MIC was read after incubation of agar plates at 37°C overnight. The MICs reported are modal values from five independent replicates. The quality control (QC) strains used were *E. coli* ATCC 25922, *E. coli* ATCC 35218, *E. coli* NCTC 13353, *K. pneumoniae* ATCC 700603, *K. pneumoniae* BAA-1705, and *K. pneumoniae* BAA-2814 and as described in the CLSI standard (5).

Spontaneous mutant selection on agar

K. pneumoniae OMAN 8 and 19 strains were grown in 20 mL of CAMHB for at least 5 hours at 37°C with shaking. Meanwhile, Mueller Hinton Agar (MHA, 1.5% final agar concentration) was prepared according to the manufacturer's directions. Autoclaved MHA was supplemented with cefepime at 4× MIC as well as taniborbactam at a fixed concentration of 4 μg/mL. The agar was aliquoted to 100 mm diameter petri dishes at 20 mL per dish. For each strain tested, 10 agar plates were made. Agar plates without drug were also made to obtain colony counts in the absence of drug pressure. For inoculum preparation, a growing culture was diluted 1:3 in a cuvette containing CAMHB and the optical density was measured at 600 nm using a Genesys 20 spectrophotometer to estimate the number of cells per sample. Cells in the culture were then concentrated by centrifugation and resuspended in 1 mL PBS to make a final suspension of approximately 1×10^{11} colony forming unit (CFU)/mL. Each agar plate received 100 µL of the cell suspension. Plates were incubated at 37°C and checked for colonies at 24 and 48 hours.

Whole genome sequencing and genome analysis

Whole genome sequencing (WGS) and genomic analyses were conducted on parent and isolated mutant strains. DNA extraction, Illumina library preparation and Illumina HiSeq 2×150 bp were

performed by GENEWIZ (South Plainfield, NJ, USA). WGS analysis from FASTQ files provided by GENEWIZ was performed using Geneious Prime version 2022.1.1 (Biomatters Inc., San Diego, CA, USA). The reads were trimmed with BBDuK Adapter/Quality Trimming Version 38.84 (Brian Bushnell), yielding ~10 million reads. De novo assembly was performed with the Geneious Assembler using ~1 million reads per genome to give 5.7 to 7.6 million nucleotides per assembly. Multi locus sequence typing was performed at the Center for Genomic Epidemiology (http://genomicepidemiology.org/services/) (6). β-Lactamases in each genome were annotated using a search set of 84 representative β-lactamases and cut-off of 40% identity. This search set successfully identifies all ~2,000 β-lactamases included in ResFinder 4.1 (7). The *ftsI* gene encoding PBP3 (the major target of cefepime) and the genes encoding major porins (OmpK36 and OmpK35) and a regulator of porins and efflux pumps (RamR) were annotated using the reference genes listed in **Table S2**. To identify genetic alterations in the genome sequences of mutants compared to that of the parent strain, the reference genome sequences were first identified using BLAST searches with an assembled genome and mapped with trimmed reads. The assembled genomes of the Kp46596 strain (NCBI ID: GCF_013694405.1) and the CFSAN054111 strain (NCBI ID: GCF_003030145.1) were identified as reference genomes for OMAN 8 and OMAN 19, respectively. Because the CFSAN054111 assembled genome does not contain the $bla_{\text{NDM-1}}$ gene encoding NDM-1, the OMAN 19 reads that were not aligned to the reference genome were mapped to the pKPM502 plasmid sequence (NCBI ID: CP031736) that contains $bla_{\text{NDM-1}}$. The average coverage of entire genome sequences and genes of NDM-1 and OXA-1 were determined as an indication of gene multiplication.

Quantification of β-lactamase activity in the cell lysate

Isolated colonies of the OMAN parent and mutant strains were grown on Muller Hinton agar at 37°C overnight and suspended in 1 mL 1× phosphate buffered saline (pH 7.4) (PBS). The cell suspension was sonicated on ice and centrifuged at 21,100 × *g* for 5 min. The supernatant (cell

lysate) was taken and used in the Bradford assay to measure the protein concentration and in the β-lactamase activity assay. In the β-lactamase activity assay, cell lysate (5 μL) was mixed with 5 μL PBS in the absence or presence of 4 μg/mL avibactam for 10 min and then the mixture (5 μL) was incubated with 100 μM nitrocefin in PBS containing 0.1 mg/mL bovine serum albumin (total reaction volume: 150 μL), followed by measurement of absorbance at 486 nm for 30 min. β-Lactamase specific activity (nanomoles of nitrocefin hydrolyzed per minute per milligram of protein) was determined using the nitrocefin extinction coefficient of 17,400 $M^{-1}cm^{-1}$.

Table S1. Mutant selection of *K. pneumoniae* strains expressing NDM-1

Strain	Agar MIC FEP + TAN* (μg/mL)	Mutant selection condition FEP + TAN (μg/mL)	Number of colonies plated	Number of selected colonies	Frequency of resistance
**OMAN 8**	4 + 4	16 + 4	1.01×10^{11}	5	4.95×10^{-11}
**OMAN 19**	4 + 4	16 + 4	0.90×10^{11}	3	3.33×10^{-11}

*Taniborbactam (TAN) was tested at a fixed concentration of 4 μg/mL in combination with cefepime (FEP).

Table S2. Reference genes from *K. pneumoniae* ATCC 13883* used in genotypic analysis

Gene name	Locus tag/CDS	Protein accession
ftsI	DR88_RS22130	WP_002888559.1
ompK35	DR88_RS17700	WP_004195943.1
ompK36	DR88_RS05545	WP_004149145.1
ramR	DR88_RS19865	WP_002893026.1

*Nucleotide accession NZ_KN046818.1.

References

1. Hamrick JC, Docquier JD, Uehara T, Myers CL, Six DA, Chatwin CL, John KJ, Vernacchio SF, Cusick SM, Trout REL, Pozzi C, De Luca F, Benvenuti M, Mangani S, Liu B, Jackson RW, Moeck G, Xerri L, Burns CJ, Pevear DC, Daigle DM. 2020. VNRX-5133 (Taniborbactam), a broad-spectrum inhibitor of serine- and metallo-beta-lactamases, restores activity of cefepime in Enterobacterales and *Pseudomonas aeruginosa*. Antimicrob Agents Chemother 64:e01963-19.
2. Dortet L, Poirel L, Al Yaqoubi F, Nordmann P. 2012. NDM-1, OXA-48 and OXA-181 carbapenemase-producing Enterobacteriaceae in Sultanate of Oman. Clin Microbiol Infect 18:E144-8.
3. Tsai YK, Liou CH, Chang FY, Fung CP, Lin JC, Siu LK. 2017. Effects of different resistance mechanisms on susceptibility to different classes of antibiotics in *Klebsiella pneumoniae* strains: a strategic system for the screening and activity testing of new antibiotics. J Antimicrob Chemother 72:3302-3316.
4. CLSI. 2018. Clinical Laboratory Standards Institute, Methods for dilution antimicrobial susceptibility tests for bacteria that grow aerobically; Approved Standards – Eleventh Edition. CLSI document M07-A11 (ISBN 1-56238-836-3). .
5. CLSI. 2021. Clinical Laboratory Standards Institute, Performance standards for antimicrobial susceptibility testing, 31st ed. CLSI supplement M100 (ISBN 978-1-68440-104-8 [Print]; ISBN 978-1-68440-105-5 [Electronic]).
6. Larsen MV, Cosentino S, Rasmussen S, Friis C, Hasman H, Marvig RL, Jelsbak L, Sicheritz-Ponten T, Ussery DW, Aarestrup FM, Lund O. 2012. Multilocus sequence typing of total-genome-sequenced bacteria. J Clin Microbiol 50:1355-61.
7. Bortolaia V, Kaas RS, Ruppe E, Roberts MC, Schwarz S, Cattoir V, Philippon A, Allesoe RL, Rebelo AR, Florensa AF, Fagelhauer L, Chakraborty T, Neumann B, Werner G, Bender JK, Stingl K, Nguyen M, Coppens J, Xavier BB, Malhotra-Kumar S, Westh H, Pinholt M,

Anjum MF, Duggett NA, Kempf I, Nykasenoja S, Olkkola S, Wieczorek K, Amaro A, Clemente L, Mossong J, Losch S, Ragimbeau C, Lund O, Aarestrup FM. 2020. ResFinder 4.0 for predictions of phenotypes from genotypes. J Antimicrob Chemother 75:3491-3500.
